# Supplementary material for: Carbon-halogen bond substitution enables high-utilization four-electron iodine redox in noncorrosive dilute electrolytes
Source: Nat Commun. 2026 Feb 21;17:3048. doi: 10.1038/s41467-026-69743-z (PMC13039508; doi:10.1038/s41467-026-69743-z)
Supplement: Supplementary file 1 — Supplementary Information [file 41467_2026_69743_MOESM1_ESM.pdf]

Supplementary Information

Carbon-Halogen Bond Substitution Enables High-Utilization Four-Electron Iodine Redox in Noncorrosive Dilute Electrolytes

Zhiheng Shi[1], Yongchao Tang[1,2*], Yue Wei[3], Guigui Liu[1], Haolong Huang[1], Jintu Qi[1], Zhenfeng Feng[1], Minghui Ye[1,2], Yufei Zhang[1,2], Zhipeng Wen[1,2], Xiaoqing Liu[1,2], Qi Yang[4], Chunyi Zhi[5*], and Cheng Chao Li[1,2*]

[1]School of Chemical Engineering and Light Industry, Guangdong University of Technology, Guangzhou, 510006, P. R. China.
[2]Guangdong Provincial Laboratory of Chemistry and Fine Chemical Engineering Jieyang Center, Jieyang, 515200, P. R. China.
[3]School of Environment and Civil Engineering, Dongguan University of Technology, Dongguan, Guangdong 523808, P. R. China.
[4]State Key Laboratory of Chemical Resource Engineering, College of Chemical Engineering, Beijing University of Chemical Technology, Beijing 100029, P. R. China
[5]Department of Materials Science and Engineering, City University of Hong Kong, Kowloon, Hong Kong, China
Email: tyc@gdut.edu.cn; cy.zhi@cityu.edu.hk; licc@gdut.edu.cn

This PDF file includes:

Supplementary Figures 1 to 60 (Pages S3-S30)
Supplementary Tables 1 to 5 (Pages S31-S33)
Supplementary References (Pages S34-S35)

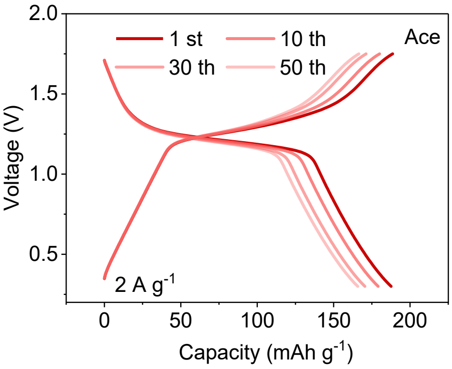

Fig. S1 Electrochemical tests of Zn||I_2 batteries. GCD curves of Zn||I_2 batteries with Ace electrolytes at different cycles.

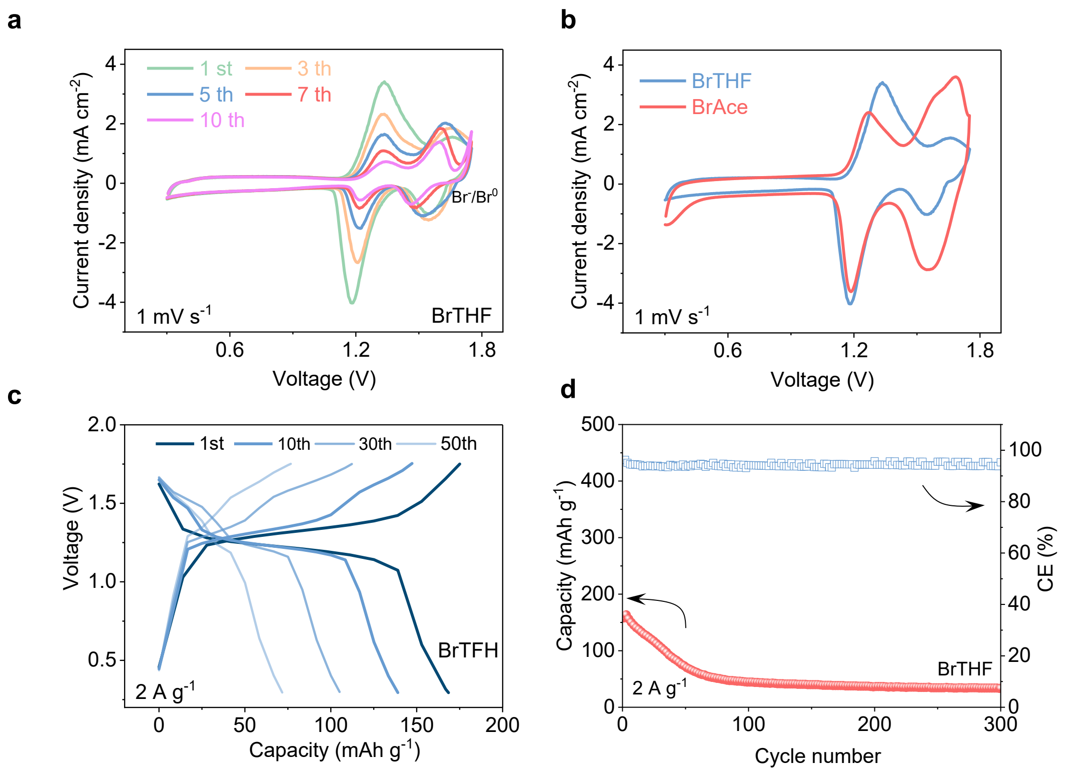

Fig. S2 Electrochemical tests of Zn||I_2 batteries. a, CV curves of Zn||I_2 batteries with BrTHF electrolyte at 1 mV s^{-1}; **b**, CV curves comparison of Zn||I_2 batteries with BrTHF and BrAce electrolyte at 1 mV s^{-1}; **c**, **d**, GCD curves and long-term cycling performance of Zn||I_2 batteries with BrTHF electrolyte at 2 A g^{-1}.

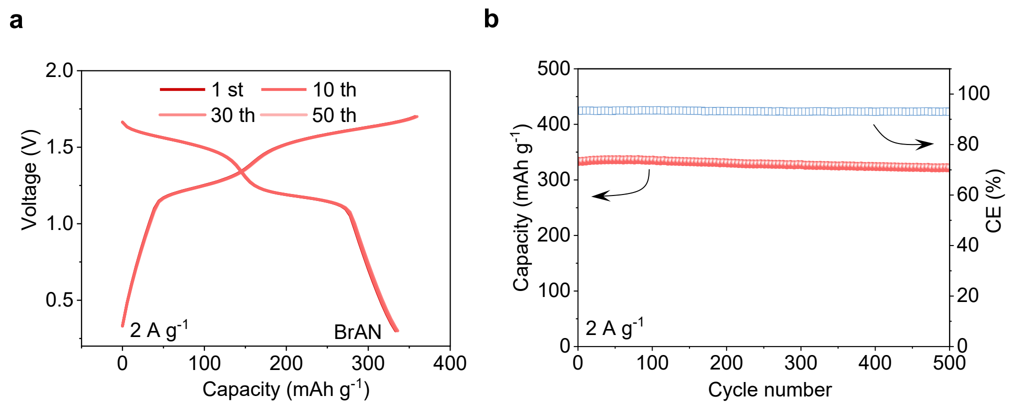

Fig. S3 Electrochemical tests of Zn||I_2 batteries. **a**, GCD curves and **b**, long-term cycling performance of Zn||I_2 batteries with BrAN electrolyte at 2 A g^{-1}.

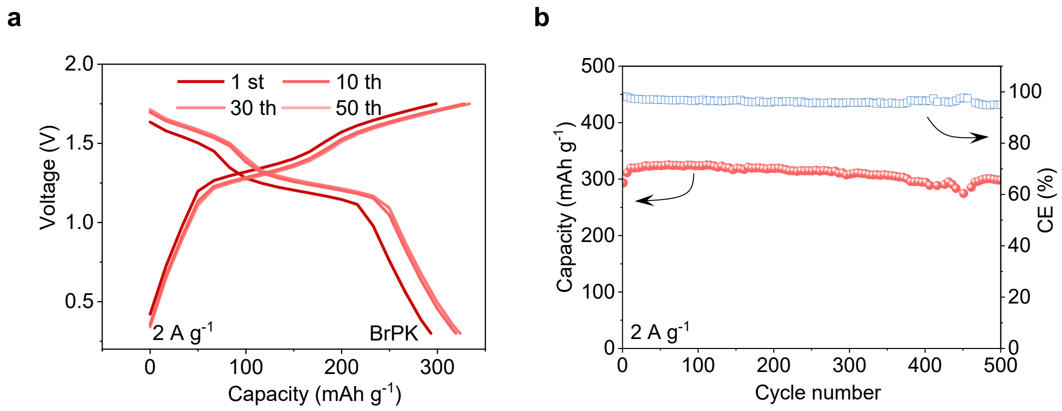

Fig. S4 Electrochemical tests of Zn||I_2 batteries. **a**, GCD curves and **b**, long-term cycling performance of Zn||I_2 batteries with BrPK electrolyte at 2 A g^{-1}.

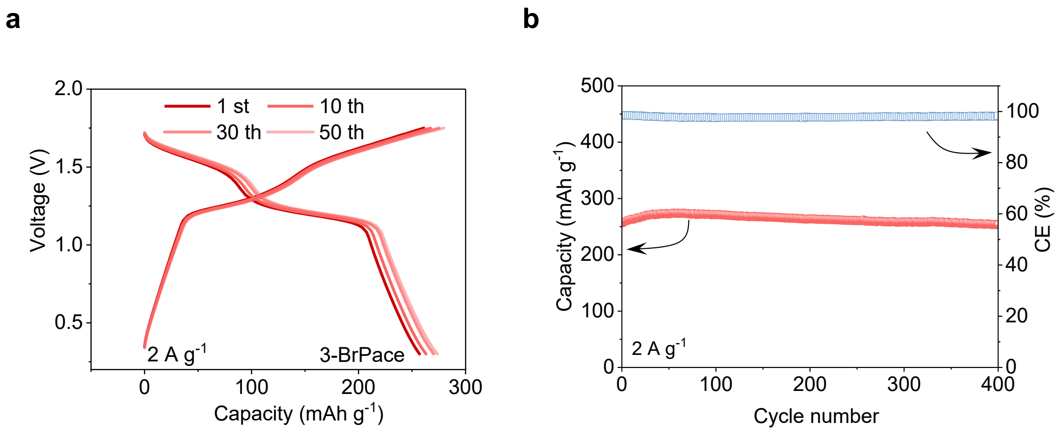

Fig. S5 Electrochemical tests of Zn||I_2 batteries. a, GCD curves and **b**, long-term cycling performance of Zn||I_2 batteries with 3-BrPace electrolyte at 2 A g^{-1}.

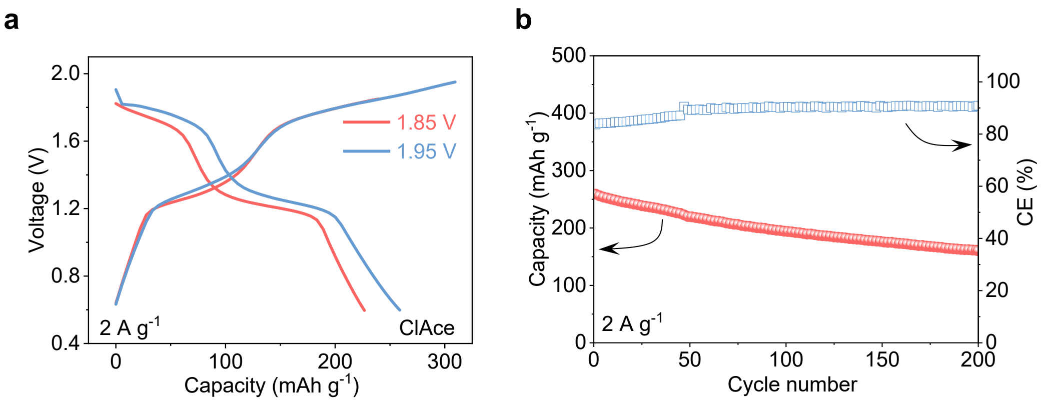

Fig. S6 Electrochemical tests of Zn||I_2 batteries. a, GCD curves for different voltage ranges of ClAce at 2 A g^{-1}. **b**, Long-term cycling performance at 2 A g^{-1} when charged to 1.85 V.

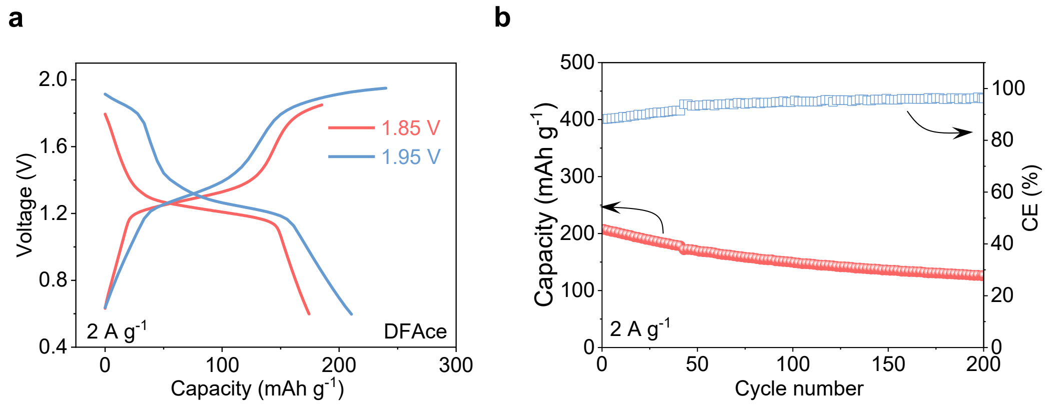

Fig. S7 Electrochemical tests of Zn||I_2 batteries. a, GCD curves of Zn||I_2 batteries in DFAce electrolytes at 2 A g^{-1}. **b**, Long-term cycling performance at 2 A g^{-1} in a cut-off voltage of 0.3-1.95 V.

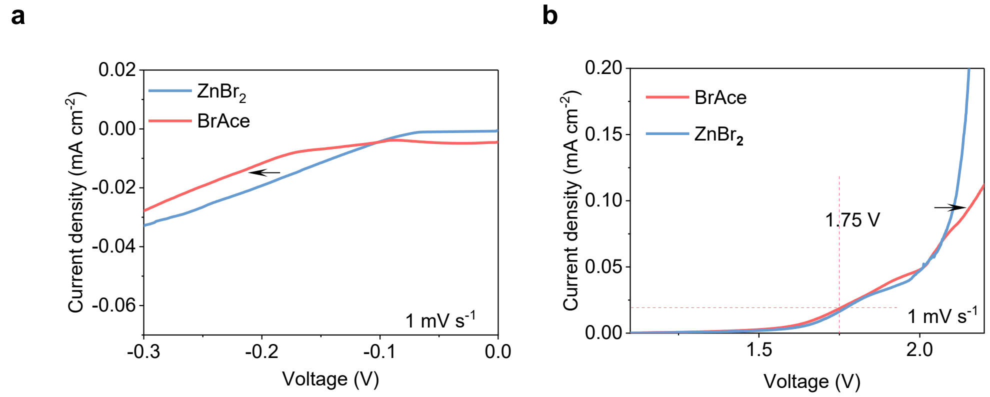

Fig. S8 LSV tests of different electrolytes. a, **b**, LSV curves of three-electrode systems (working electrode: Zn; reference electrode: Ag/AgCl; counter electrode: Pt) with $ZnBr_2$ and BrAce electrolytes.

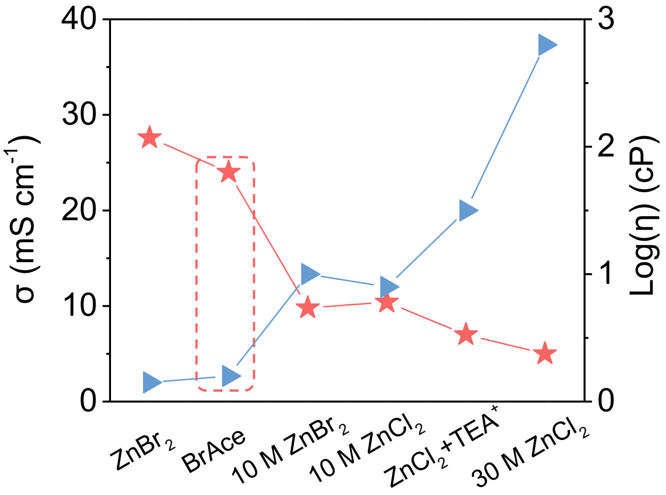

Fig. S9 Ionic conductivity-viscosity tests of different electrolytes. Ionic conductivity (red) and viscosity (blue) of electrolytes for $I^-/I^0/I^+$ redox[1-4].

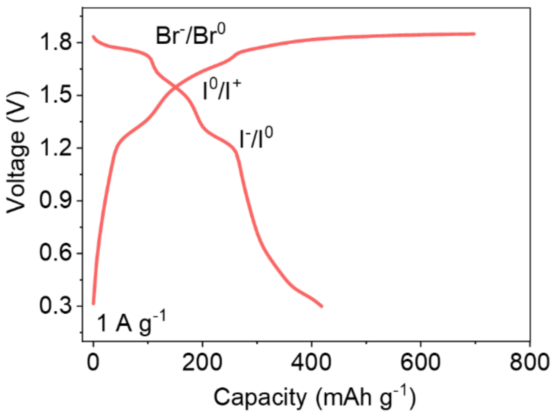

Fig. S10 Electrochemical test in a cutoff voltage of 0.3–1.85 V. GCD curve of a Zn||I_2 battery assembled with BrAce electrolyte charged to 1.85 V at a specific current of 1 A g^{-1}.

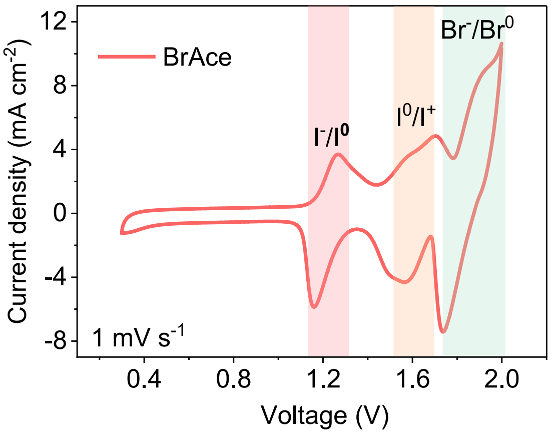

Fig. S11 Electrochemical test of Zn||I$_2$ batteries. CV curve of Zn||I$_2$ batteries with BrAce electrolytes in a cut-off voltage of 0.3-2.0 V.

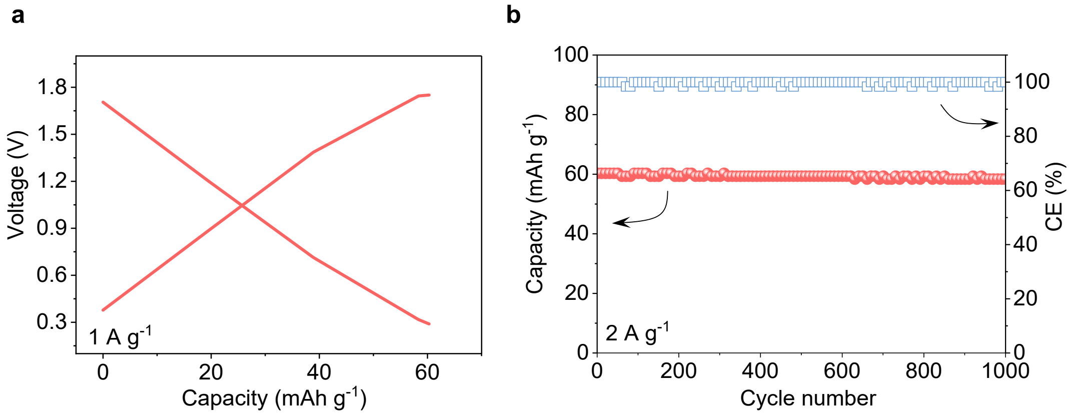

Fig. S12 Electrochemical tests of iodine-free battery. a, GCD curve of the iodine-free battery assembled with BrAce electrolyte at a specific current of 1 A g^{-1}. **b**, The corresponding long-term cycling performance.

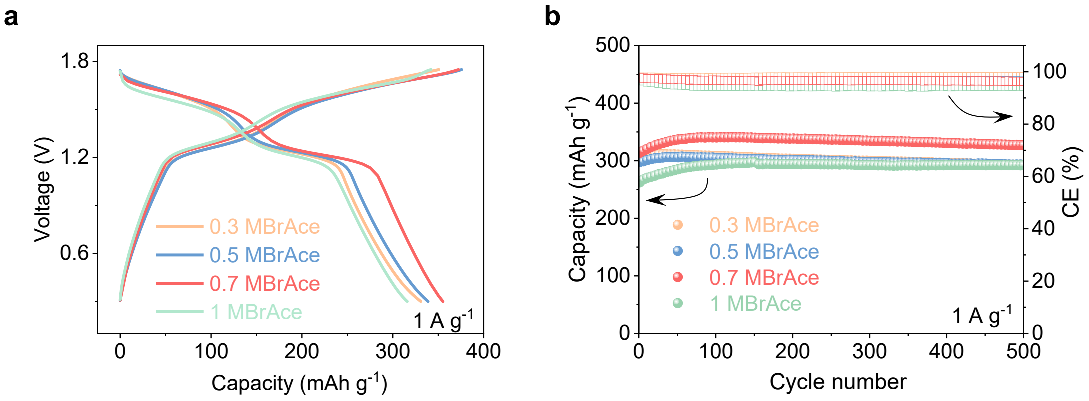

Fig. S13 Electrochemical tests of Zn||I_2 batteries. a, GCD curves and **b**, long-term cycling performance of Zn||I_2 batteries in electrolytes with different BrAce concentrations at 1 A g^{-1}.

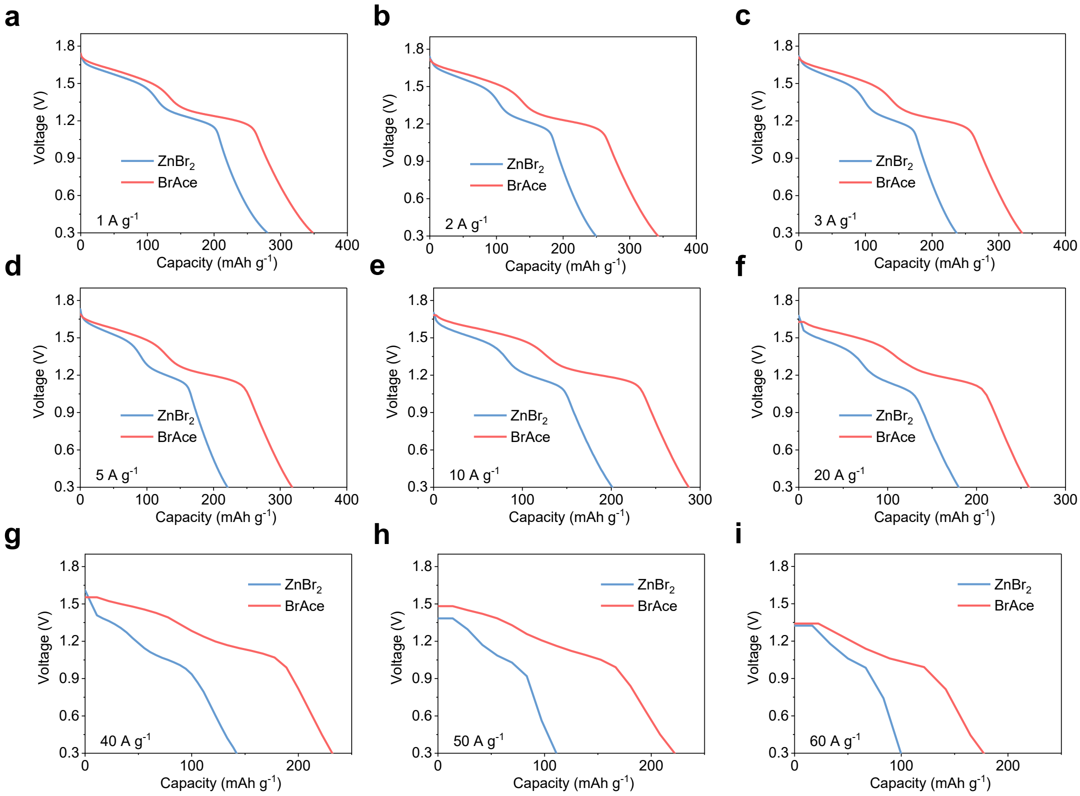

Fig. S14 Discharge curves at different rates. a-i, Discharge curves of Zn||I_2 batteries at ultrahigh rates of 1-60 A g^{-1} with BrAce and $ZnBr_2$ electrolytes.

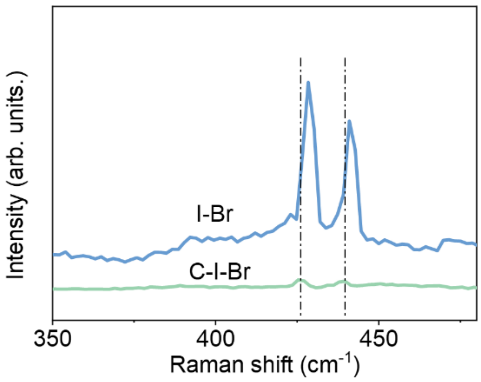

Fig. S15 Raman spectroscopy tests. Raman spectra of I-Br and C-I-Br intermediates.

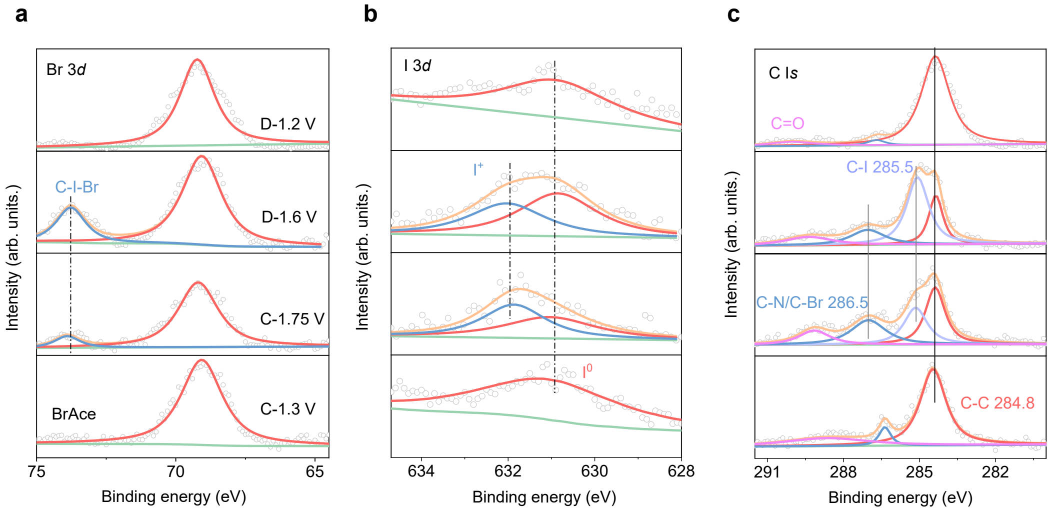

Fig. S16 XPS analysis of positive electrodes. Ex situ XPS spectra of positive electrodes during GCD in BrAce electrolyte: **a**, Br 3*d* spectra; **b**, I 3*d* spectra; **c**, C 1*s* spectra.

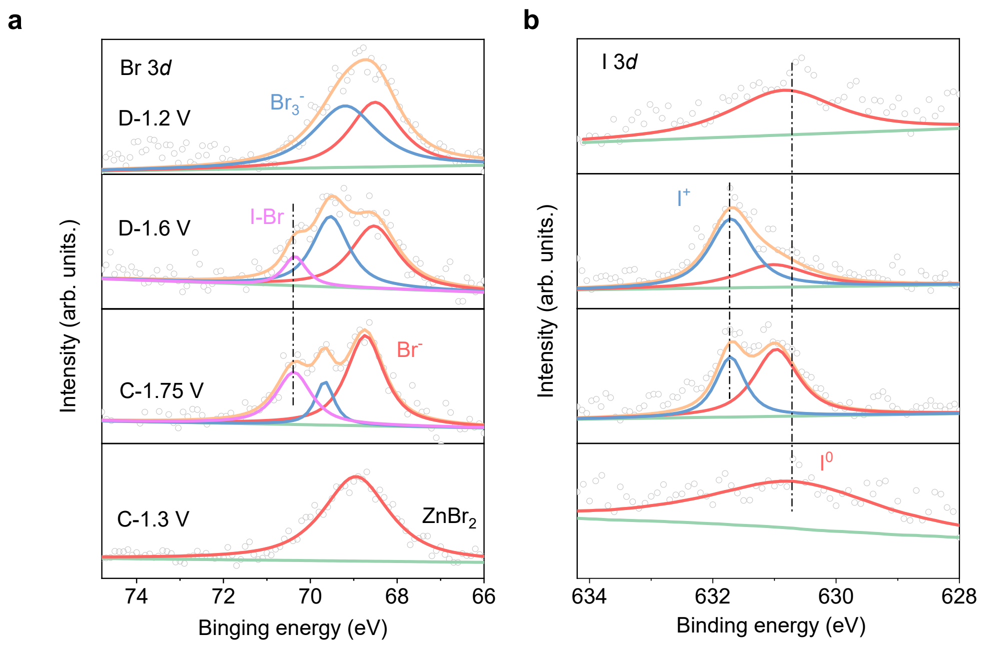

Fig. S17 XPS analysis of positive electrodes. Ex situ XPS spectra of positive electrodes during GCD in $ZnBr_2$ electrolyte: **a**, Br 3*d* spectra; **b**, I 3*d* spectra.

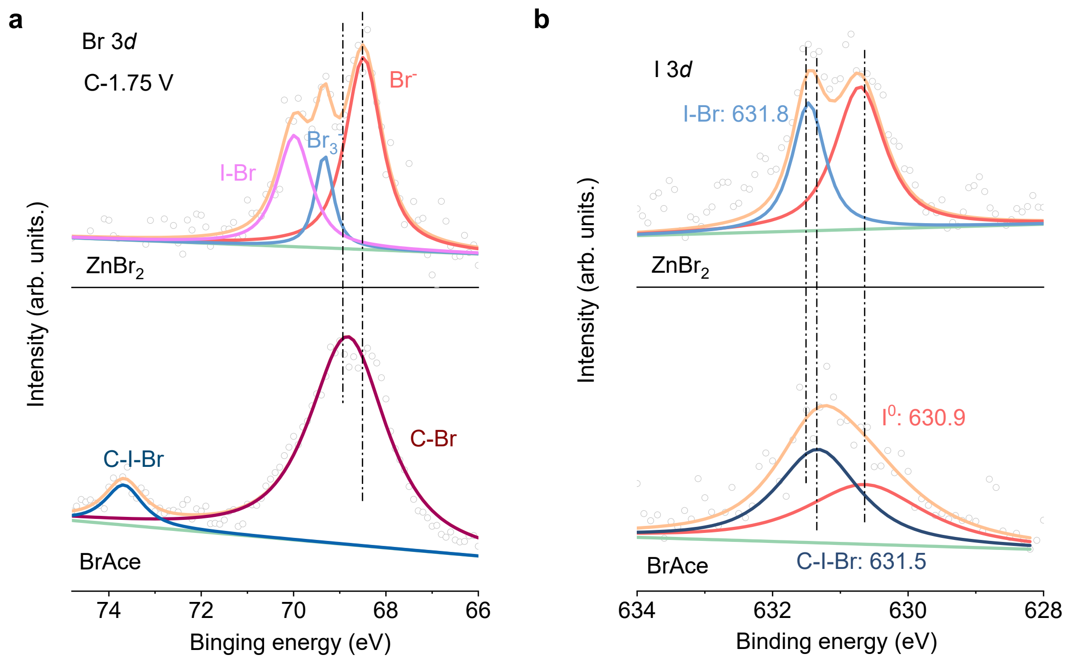

Fig. S18 XPS analysis of positive electrodes. Ex situ XPS spectra of the positive electrodes charged to 1.75 V in $ZnBr_2$ and BrAce electrolytes: **a**, Br 3*d* spectra; **b**, I 3*d* spectra.

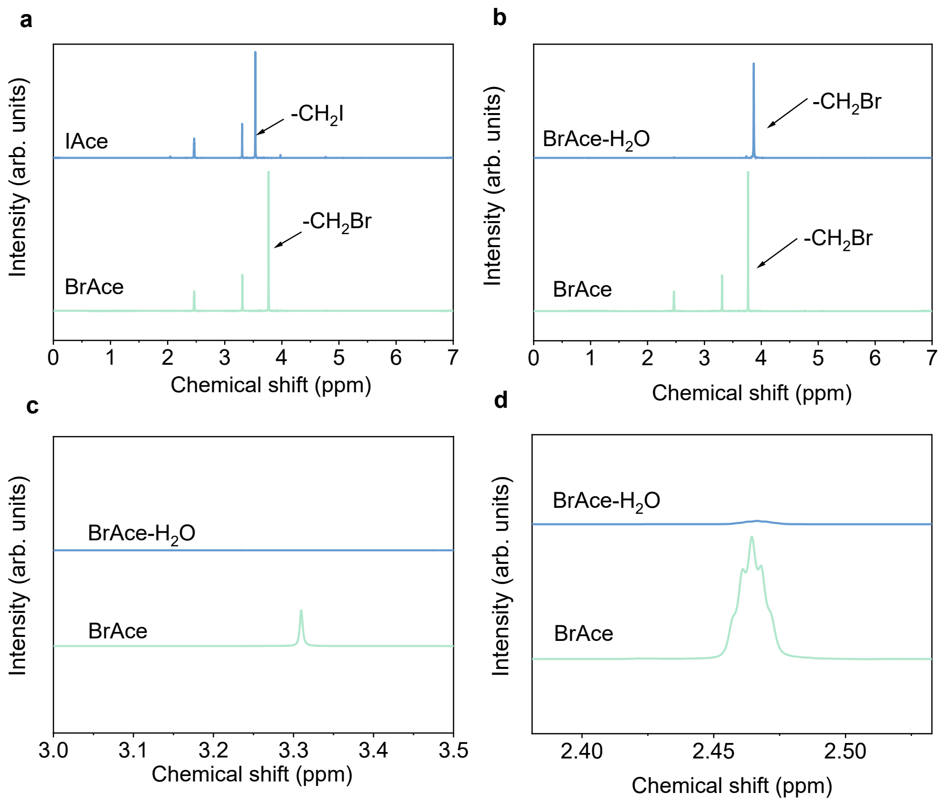

Fig. S19 NMR analysis of different electrolytes. a, ^{1}H NMR spectra of BrAce and IAce. **b-d**, ^{1}H NMR spectra of BrAce and BrAce in deionized water.

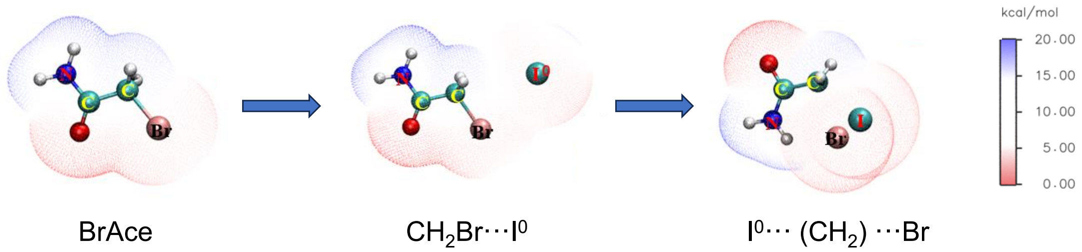

Fig. S20 Electrostatic potential calculation of different structures. Electrostatic potential in different reaction states, including BrAce, $CH_2Br\cdots I^0$, and the $I^0\cdots(CH_2)\cdots Br$. Red spheres: O atoms; Blue spheres: N atoms; Brown spheres: Br atoms; Dark green spheres: I atoms; Light green spheres: C atoms; White spheres: H atoms.

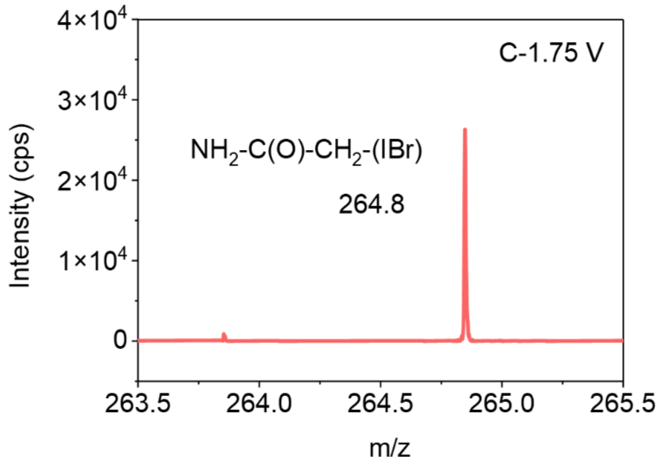

Fig. S21 Mass spectrometry analysis. Mass spectrometry of C-I-Br sample (C-1.75 V).

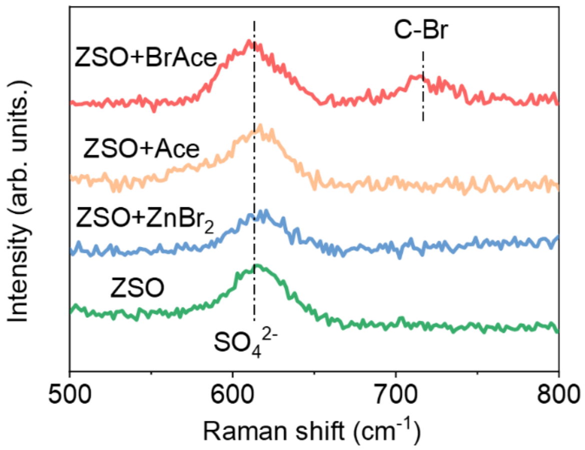

Fig. S22 Raman spectrum analysis of different electrolytes. Raman spectra of ZSO, $ZSO+ZnBr_2$, ZSO+Ace, and ZSO+BrAce electrolytes.

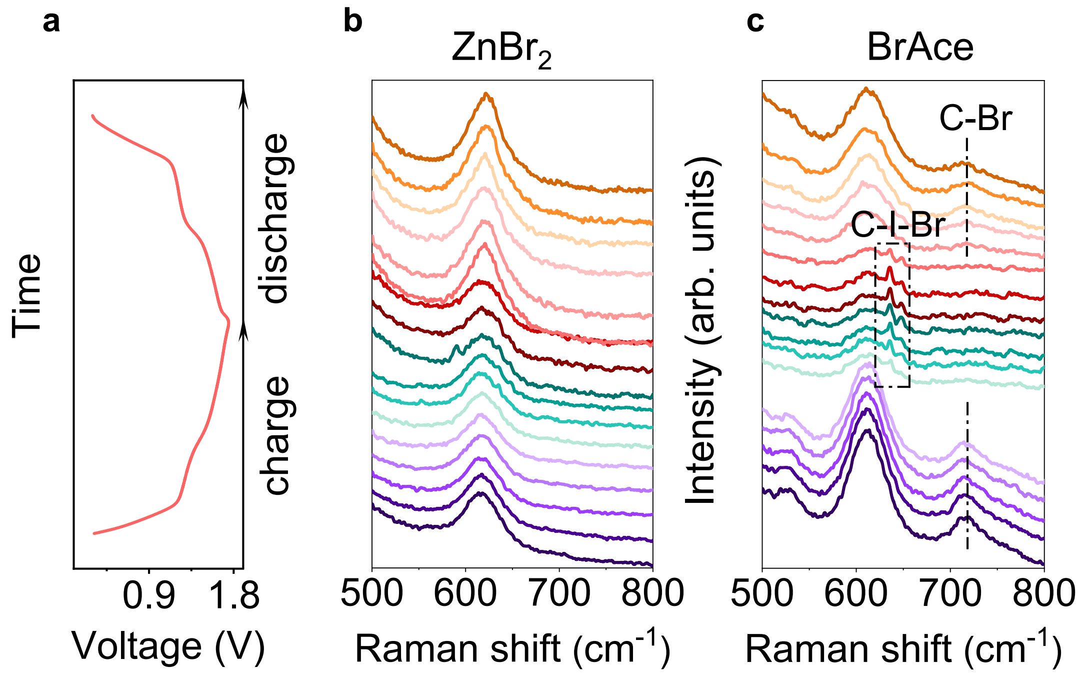

Fig. S23 In situ Raman spectrum analysis of positive electrodes. a, Typical charge/discharge curves. **b**, **c**, Corresponding in situ Raman spectra of positive electrodes in 0.35 M $ZnBr_2$ and 0.7 M BrAce electrolytes.

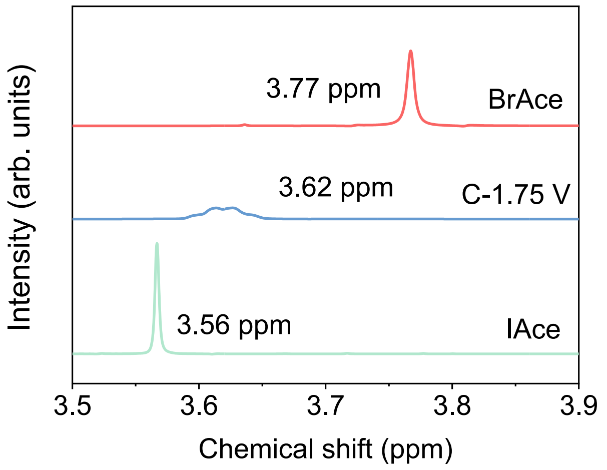

Fig. S24 NMR spectrum analysis. ^{1}H NMR spectra of IAce, C-I-Br sample (C-1.75 V), and IAce.

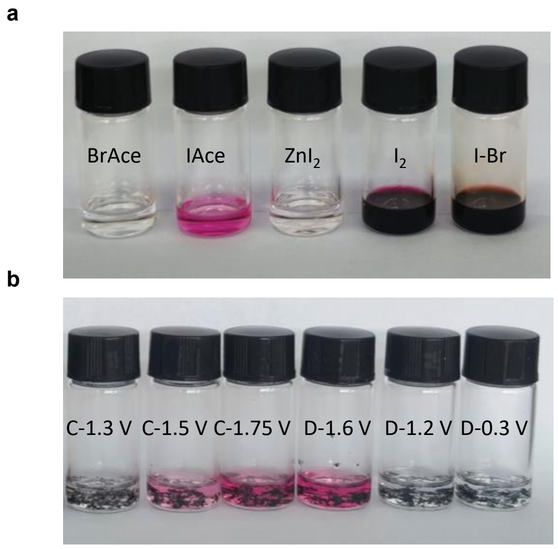

Fig. S25 Photographs of different iodine products. a, Photographs for BrAce, IAce, and different iodides dissolved in $CDCl_3$. **b**, Photographs for the products of the iodine positive electrodes at different potentials dissolved in $CDCl_3$.

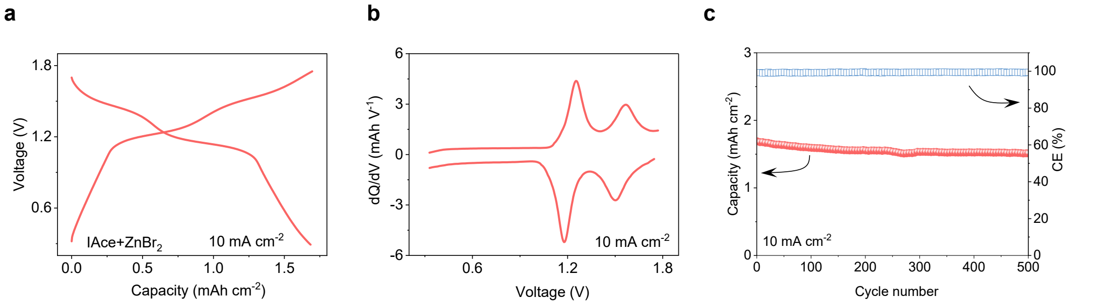

Fig. S26 Electrochemical tests of Zn||I_2 batteries with IAce+$ZnBr_2$ electrolyte. a, GCD curves; **b**, corresponding differential charge-discharge curves; **c**, corresponding long-term cycling performance.

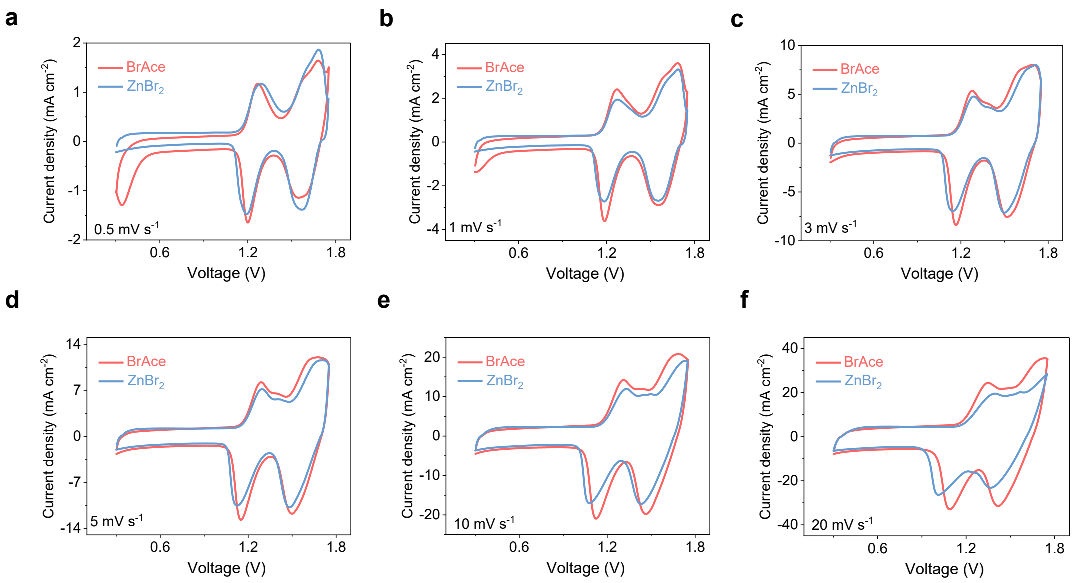

Fig. S27 Comparison of different CV curves. a-e, CV curves of Zn||I2 batteries with BrAce and $ZnBr_2$ electrolytes at different scan rates.

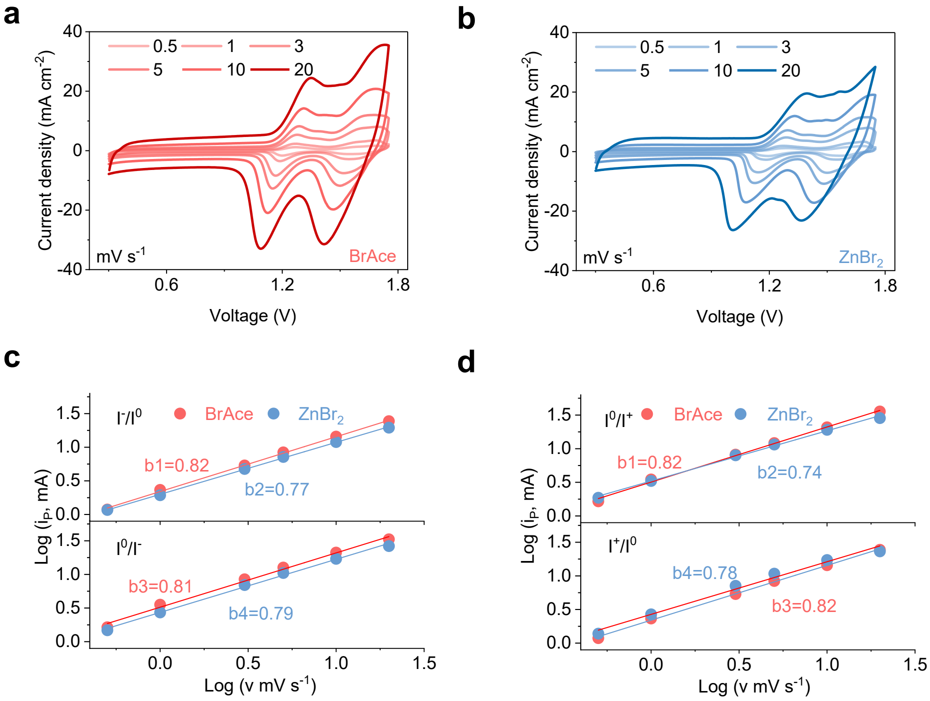

Fig. S28 CV tests and analysis. a, **b**, CV curves of Zn||I2 batteries with BrAce and $ZnBr_2$ electrolytes at scan rates of 1-20 mV s^{-1}. **c**, **d**, Logi versus log*ν* plots of the current response with BrAce and $ZnBr_2$ electrolytes.

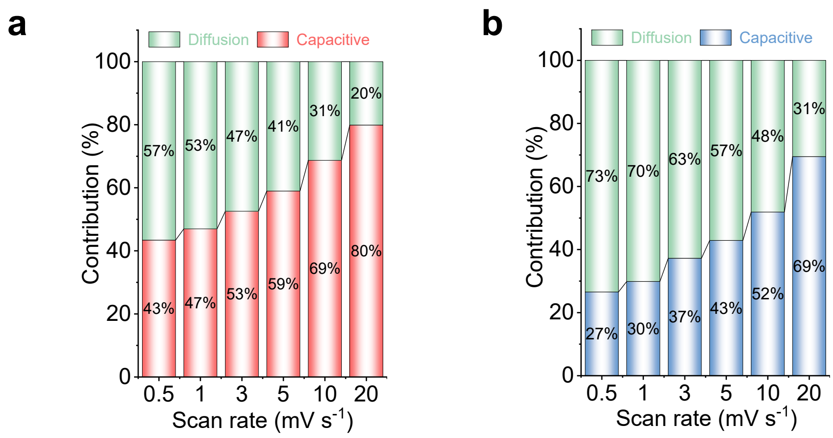

Fig. S29 Capacity contribution analysis of Zn||I_2 batteries. Diffusion-controlled and capacitive contributions of Zn||I_2 batteries at different scan rates: **a**, with BrAce electrolyte; **b**, with $ZnBr_2$ electrolyte.

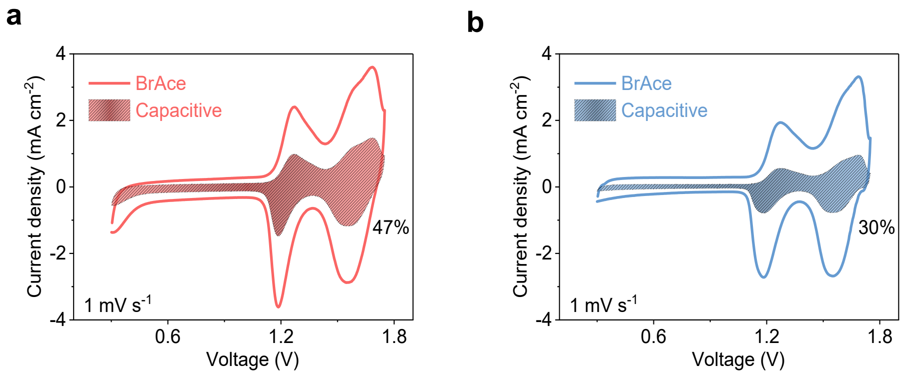

Fig. S30 Capacity contribution proportion of Zn||I_2 batteries. a, CV curves and corresponding capacitive contribution (47%, shaded part) of Zn||I_2 batteries with BrAce electrolyte at 1 mV s^{-1}; **b**, CV curves and corresponding capacitive contribution (30%, shaded part) of Zn||I_2 batteries with $ZnBr_2$ electrolyte at 1 mV s^{-1}.

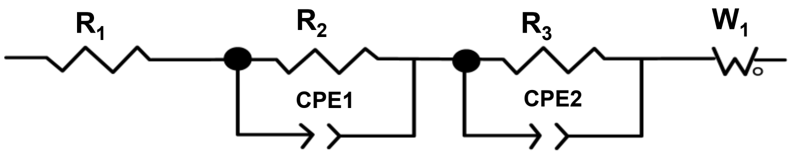

Fig. S31 Equivalent circuit diagram. The equivalent circuit of the EIS fitting. R_1 is the intersection of the diagram with the real axis refers to a bulk resistance; R_2 is the interfacial resistance, R_3 is the charge transfer resistance; C is the capacitor and W_1 is the Warburg element.

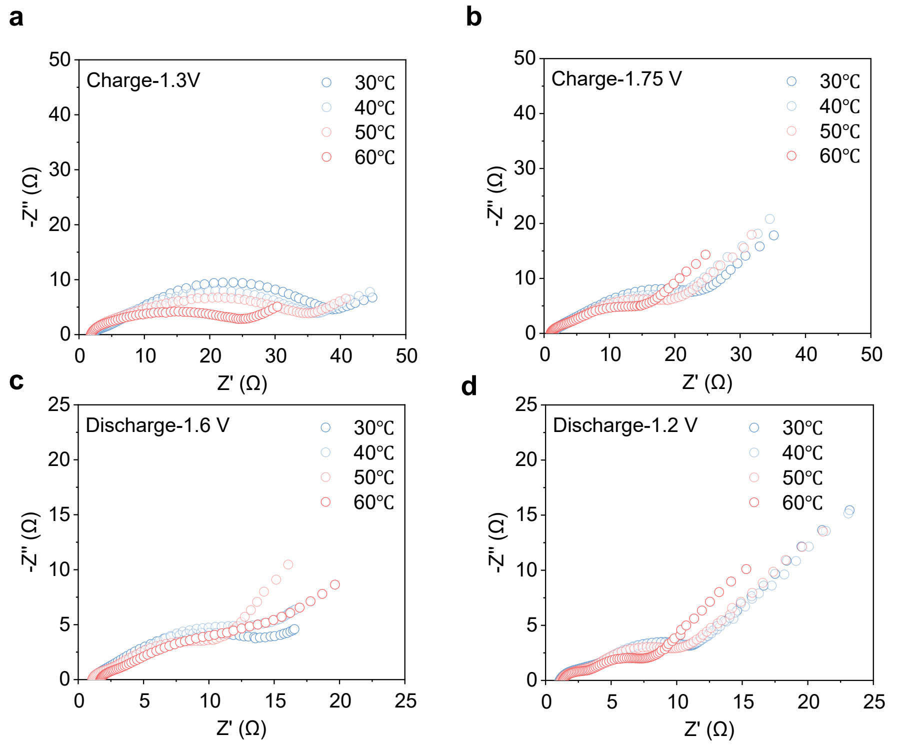

Fig. S32 Variable-temperature EIS tests. a-d, EIS spectra of Zn||I_2 batteries with BrAce electrolytes at different temperatures and charged/discharged states.

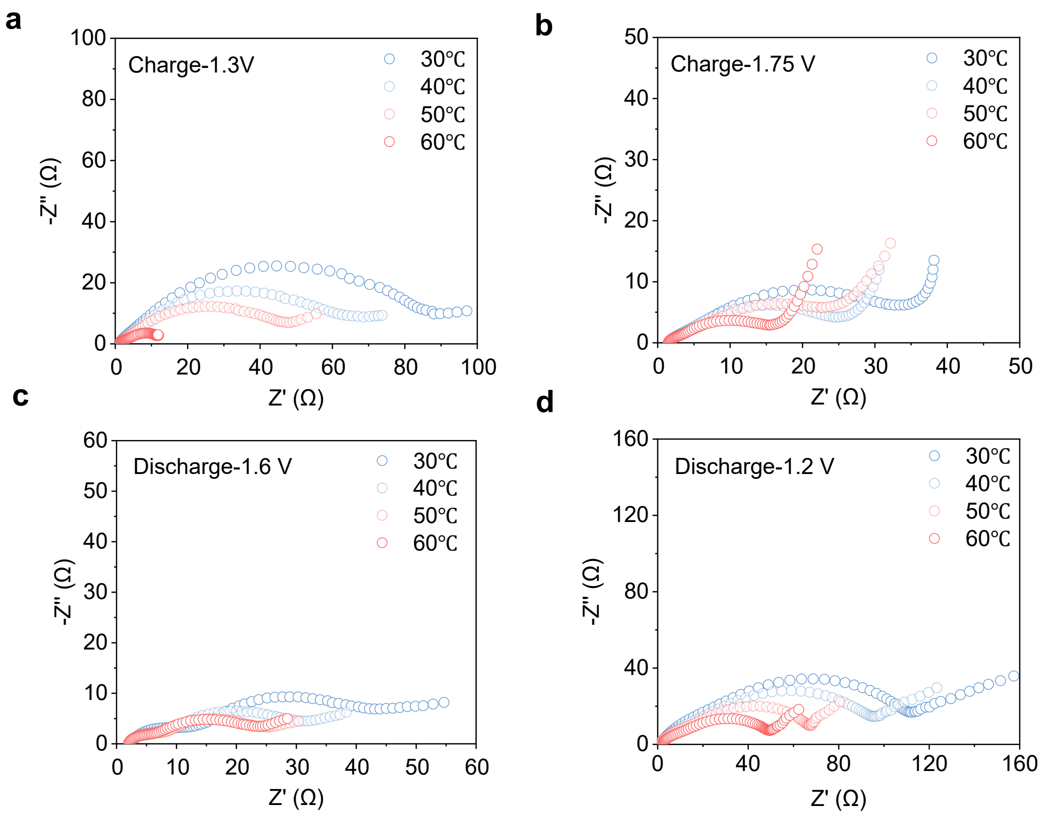

Fig. S33 Variable-temperature EIS tests. a-d, EIS spectra of Zn||I_2 batteries with $ZnBr_2$ electrolytes at different temperatures and charged/discharged states.

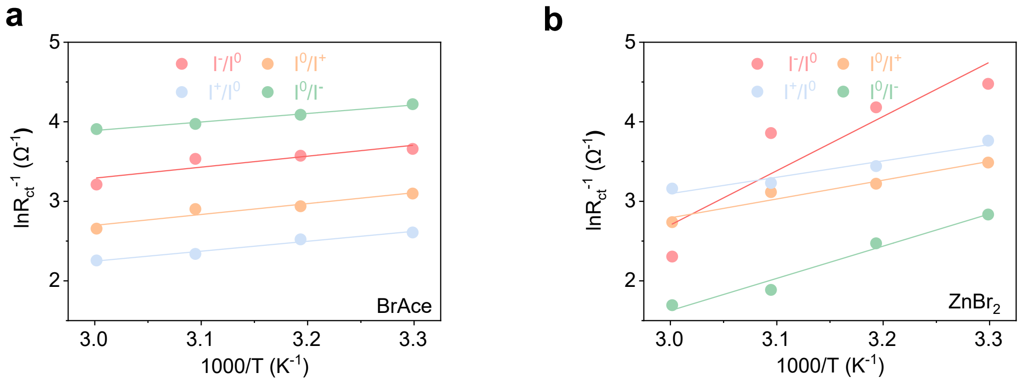

Fig. S34 Arrhenius plots analysis. Arrhenius plots of Zn||I_2 batteries with **a**, BrAce and **b**, $ZnBr_2$ electrolytes at different temperatures and charged/discharged states.

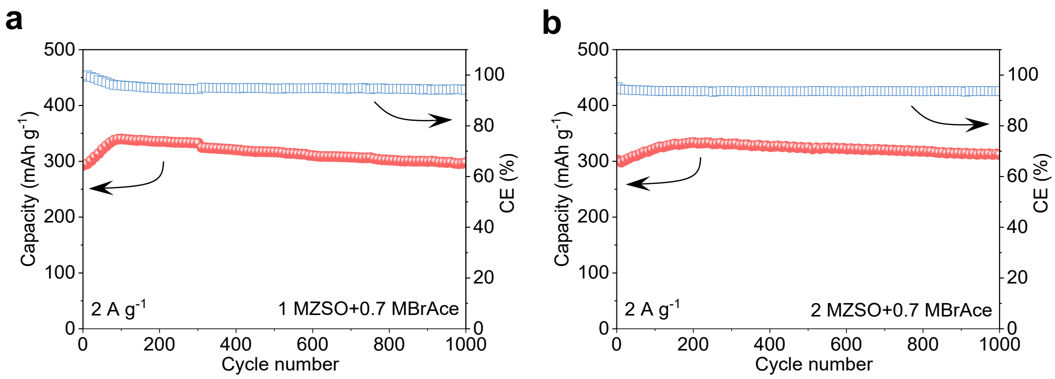

Fig. S35 Electrochemical tests of Zn||I_2 batteries. Cycling performance of Zn||I_2 batteries in **a**, 1 M ZSO and **b**, 2 M ZSO electrolytes at 2 A g^{-1}.

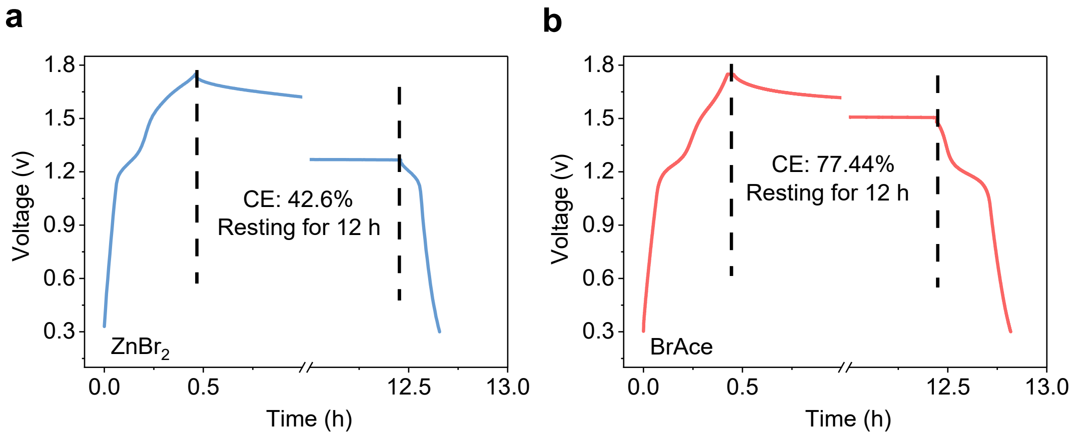

Fig. S36 Self-discharge tests of Zn||I_2 batteries. Self-discharge curves using **a**, $ZnBr_2$ and **b**, BrAce electrolytes after standing for 12 h in fully charged state.

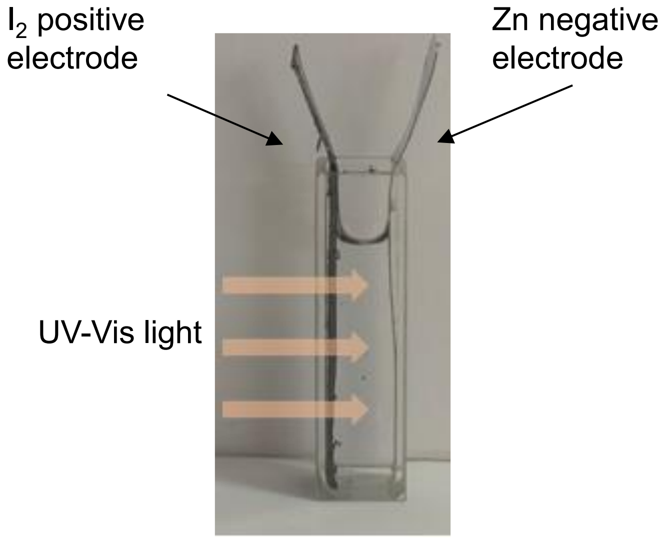

Fig. S37 Photograph of a cuvette cell. The cuvette cell for in situ UV-Vis spectral characterization.

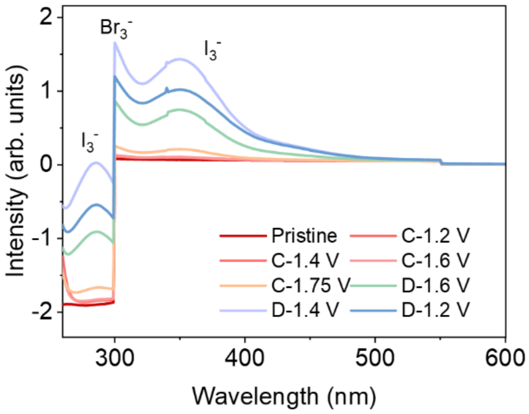

Fig. S38 UV-Vis spectroscopy tests. In situ UV-Vis spectra of I_2 positive electrode in Zn||I_2 batteries using $ZnBr_2$ electrolyte at different charging/discharging states.

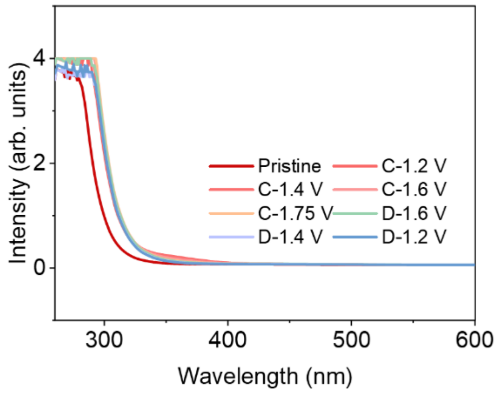

Fig. S39 UV-Vis spectroscopy tests. In situ UV-Vis spectra of I_2 positive electrode in Zn||I_2 batteries using BrAce electrolytes at different charging/discharging states.

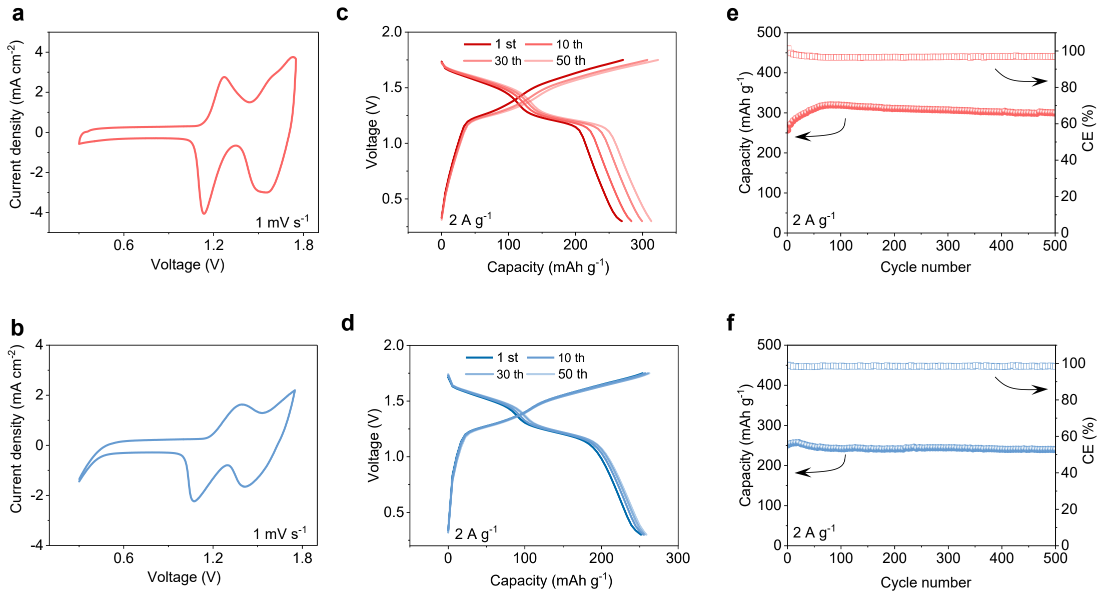

Fig. S40 Electrochemical tests of Zn||I_2 batteries. a, **b**, CV curves of Zn||I_2 batteries in 2-Bromo-N-methylacetamide and 2-Bromo-N, N-dimethylacetamide electrolytes at 1 mV s^{-1}. **c**, **d**, GCD profiles for 50 cycles. **e**, **f**, Long-term cycling performance at 2 A g^{-1}.

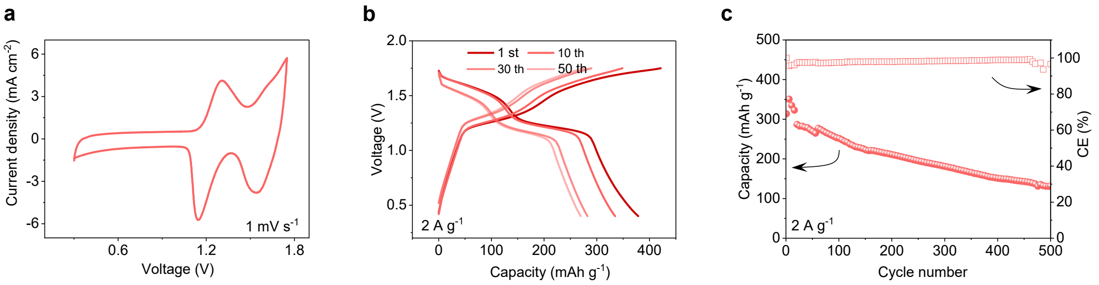

Fig. S41 Electrochemical tests of Zn||I₂ batteries. **a**, CV curves of Zn||I₂ batteries in N-bromoacetamide electrolyte at 1 mV s^{-1}. **b**, GCD profiles for different cycles. **c**, Cycling performance at 2 A g^{-1}.

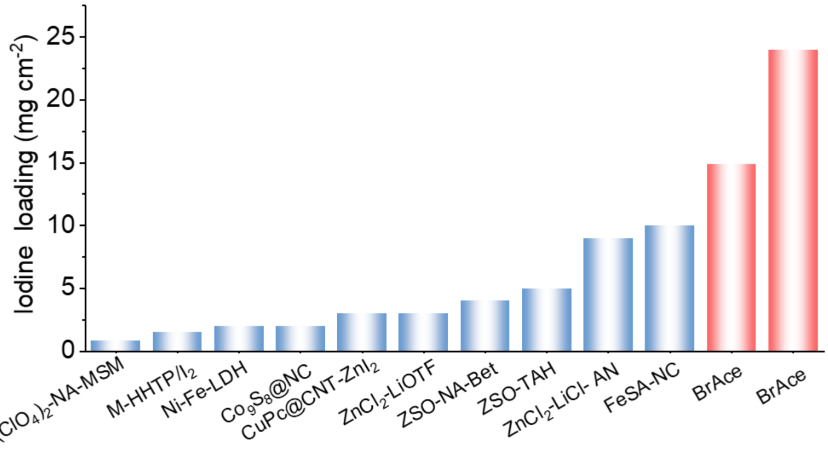

Fig. S42 Iodine loading comparison of different studies. I_2 loadings for the investigation of $I^-/I^0/I^+$ redox reported previously[2, 5-13].

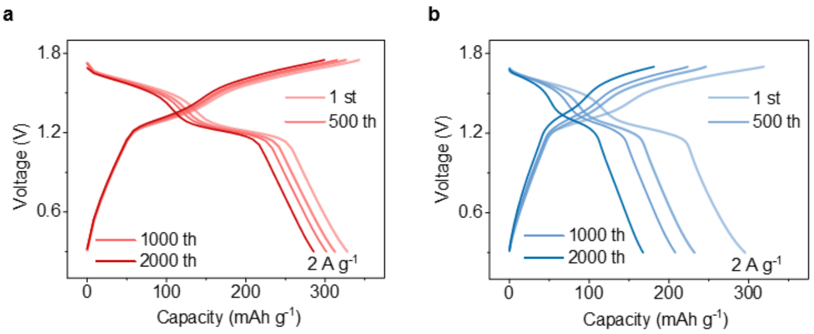

Fig. S43 Electrochemical tests of Zn||I₂ batteries. GCD curves of Zn||I₂ batteries at a current density of 2 A g^{-1}: **a**, BrAce and **b**, $ZnBr_2$ electrolytes, respectively.

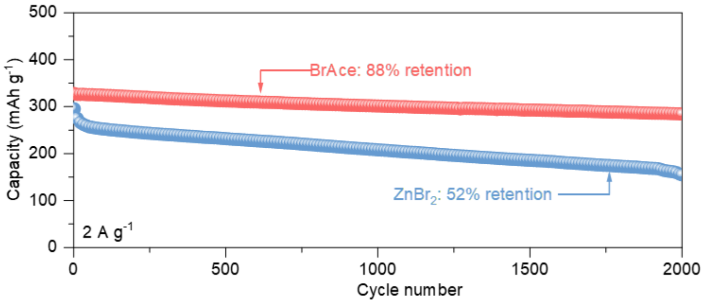

Fig. S44 Electrochemical tests of Zn‖I_2 batteries. The cycling performance of Zn||I_2 batteries in $ZnBr_2$ and BrAce electrolytes at 2 A g^{-1}.

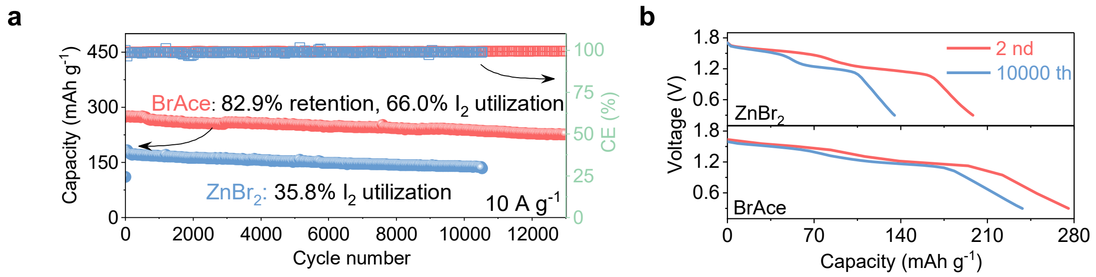

Fig. S45 Electrochemical tests of Zn‖I_2 batteries. a, Long-term cycling performance and **b**, discharge curves of Zn||I_2 batteries (I_2 loading: 3.0 mg cm^{-2}) with BrAce electrolyte and $ZnBr_2$ electrolyte at 10 A g^{-1}.

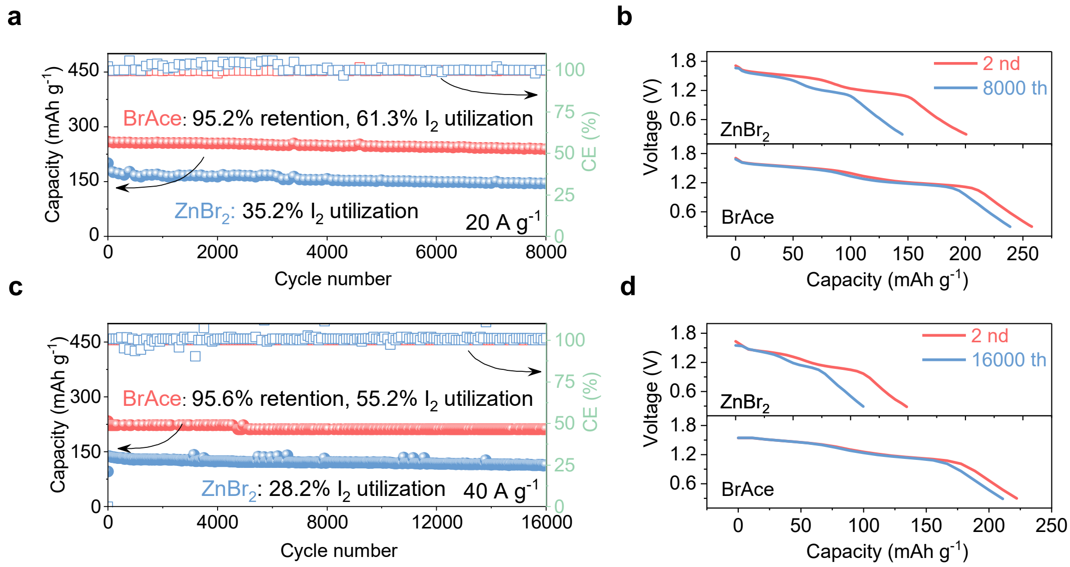

Fig. S46 Electrochemical tests of Zn‖I_2 batteries. **a**, Long-term cycling performance and **b**, discharge curves of Zn‖I_2 batteries (I_2 loading: 3.0 mg cm^{-2}) with BrAce electrolyte and $ZnBr_2$ electrolyte at 20 A g^{-1}; **c**, Long-term cycling performance and **d**, discharge curves of Zn‖I_2 batteries (I_2 loading: 3.0 mg cm^{-2}) with BrAce electrolyte and $ZnBr_2$ electrolyte at 40 A g^{-1}.

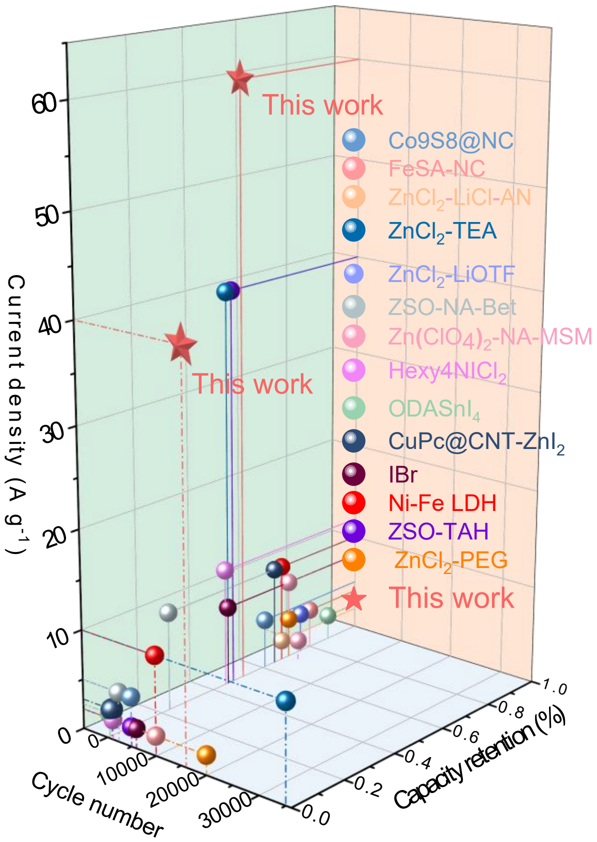

Fig. S47 Comprehensive performance comparison. Rate capability and long-term cycling performance comparison of Zn||I_2 batteries with different electrolytes[2, 3, 5, 7-17].

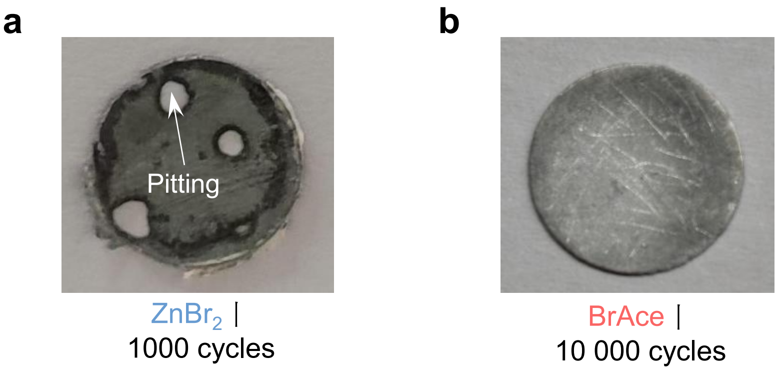

Fig. S48 Photographs of Zn negative electrodes cycled. Zn negative electrodes cycled in Zn||I_2 batteries: **a**, 1000 cycles with $ZnBr_2$ electrolyte; **b**, 10 000 cycles with BrAce electrolyte.

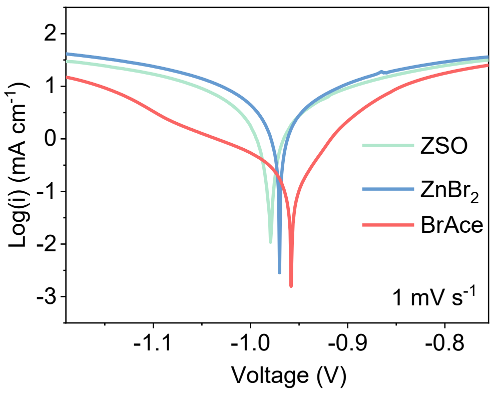

Fig. S49 Tafel curve tests. Tafel curves of Zn metal in ZSO, $ZnBr_2$, and BrAce electrolytes.

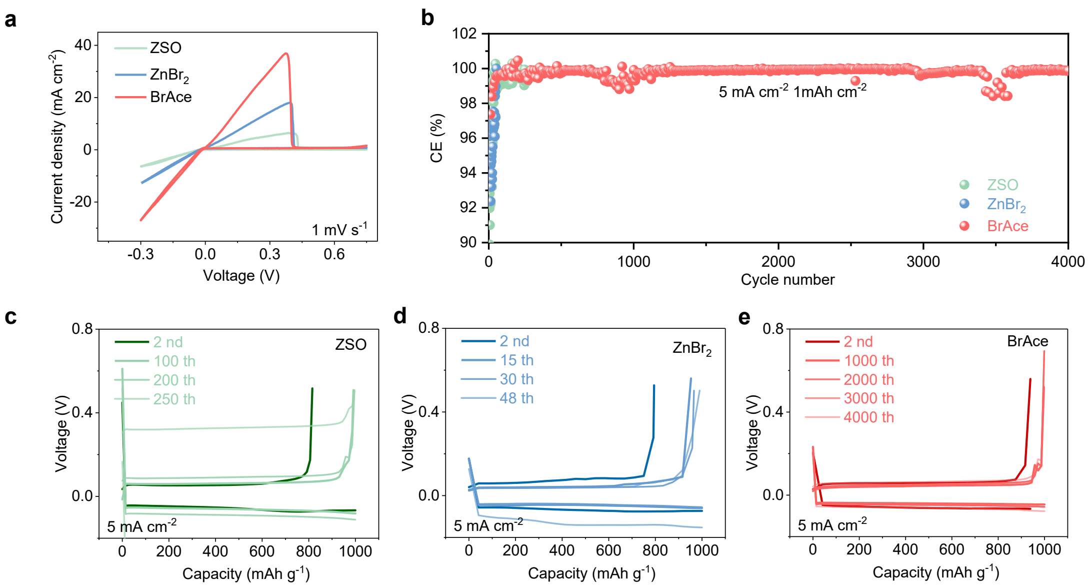

Fig. S50 Electrochemical tests of Zn‖Cu asymmetric cells. a, CV curves of Zn‖Cu asymmetric cells at a scan rate of 1 mV s^{-1} in different electrolytes. **b**, Zn plating/stripping performance of Zn‖Cu asymmetric cells at 5 mA cm^{-2} with 1 mA h cm^{-2} in different electrolytes. **c-e**, Charge/discharge profiles corresponding to Zn‖Cu asymmetric cells in different electrolytes.

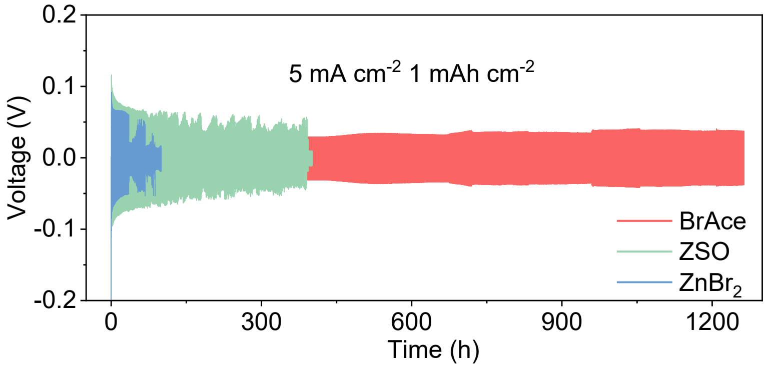

Fig. S51 Long-term cycling performance of Zn||Zn symmetric cells. Plating/stripping performance of Zn||Zn symmetric batteries using ZSO, $ZnBr_2$ and BrAce electrolytes at current density of 5 mA cm^{-2} with accumulative capacity of 1 mAh cm^{-2}.

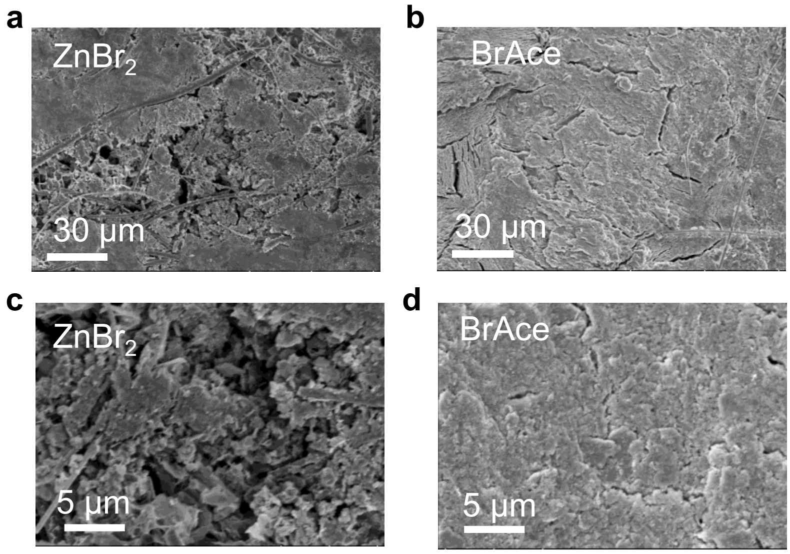

Fig. S52 SEM images of Zn negative electrodes cycled. SEM images of Zn negative electrodes after 50 cycles in Zn||I_2 batteries with **a**, **c**, $ZnBr_2$ electrolyte and **b**, **d**, BrAce electrolytes.

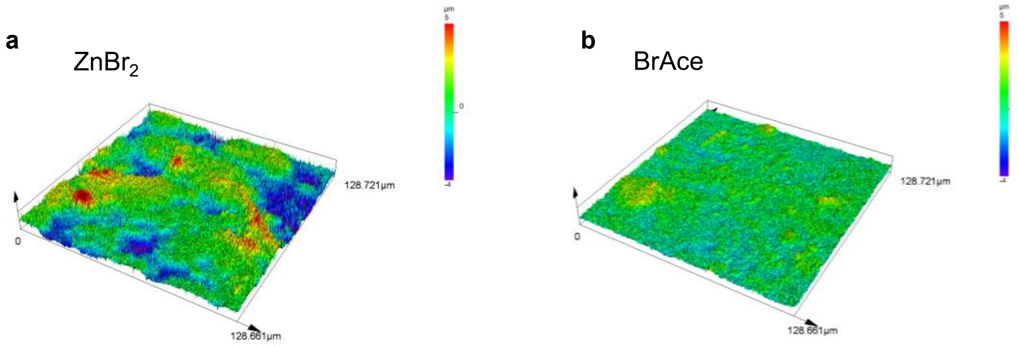

Fig. S53 LSCM images of Zn negative electrodes cycled. LSCM images of Zn negative electrodes in Zn||I_2 batteries (after 50 cycles) with **a**, $ZnBr_2$ electrolyte and **b**, BrAce electrolyte.

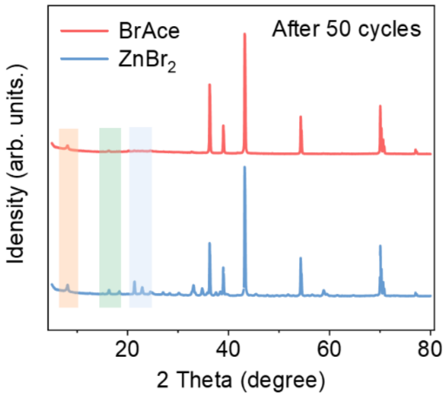

Fig. S54 Surficial passivation analysis of Zn negative electrodes. XRD patterns of Zn negative electrodes of Zn||I_2 batteries cycled in $ZnBr_2$ and BrAce electrolytes.

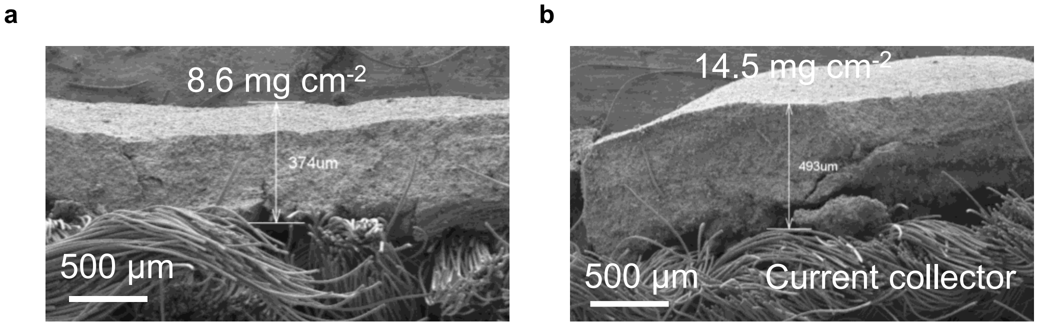

Fig. S55 Electrode thickness measurements by SEM. SEM images of electrodes with different I_2 loadings: **a**, 8.6 mg cm^{-2}; **b**, 14.5 mg cm^{-2}.

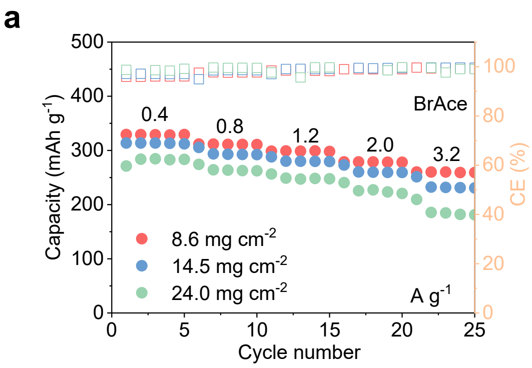

Fig. S56 Electrochemical tests of Zn‖I_2 batteries. Rate capability of Zn‖I_2 batteries with different I_2 loadings (8.6, 14.5, and 24.0 mg cm^{-2}) in **a**, BrAce electrolyte and **b**, $ZnBr_2$ electrolyte.

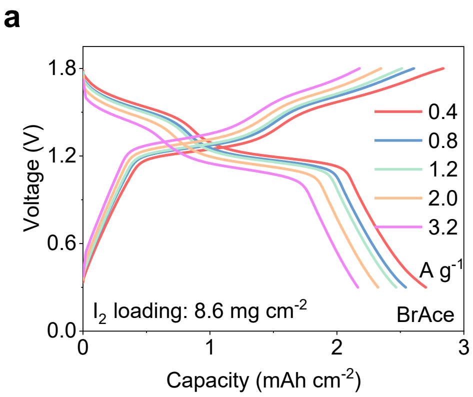

Fig. S57 Electrochemical tests of Zn‖I_2 batteries. GCD curves corresponding to rate capability of Zn‖I_2 batteries (I_2 loading: ~8.6 mg cm^{-2}) with **a**, BrAce electrolyte and **b**, $ZnBr_2$ electrolyte.

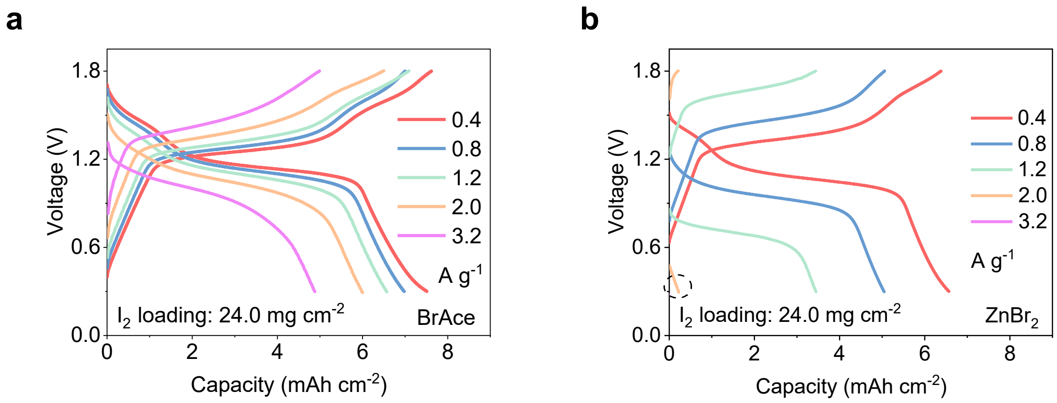

Fig. S58 Electrochemical tests of Zn||I_2 batteries. GCD curves corresponding to rate capability of Zn||I_2 batteries (I_2 loading: ~24.0 mg cm^{-2}) with **a**, BrAce electrolyte and **b**, $ZnBr_2$ electrolyte.

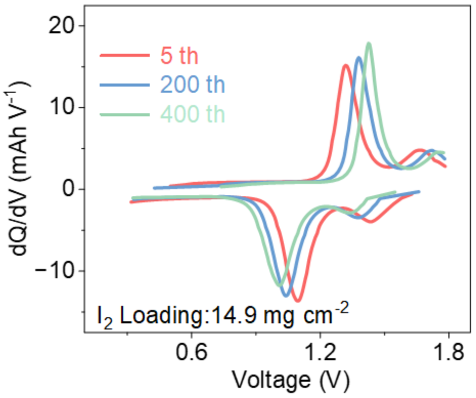

Fig. S59 Differential charge/discharge analysis. Differential charge/discharge profiles of Zn||I_2 batteries with BrAce electrolyte corresponding to the GCD cycling in **Fig. 5f**.

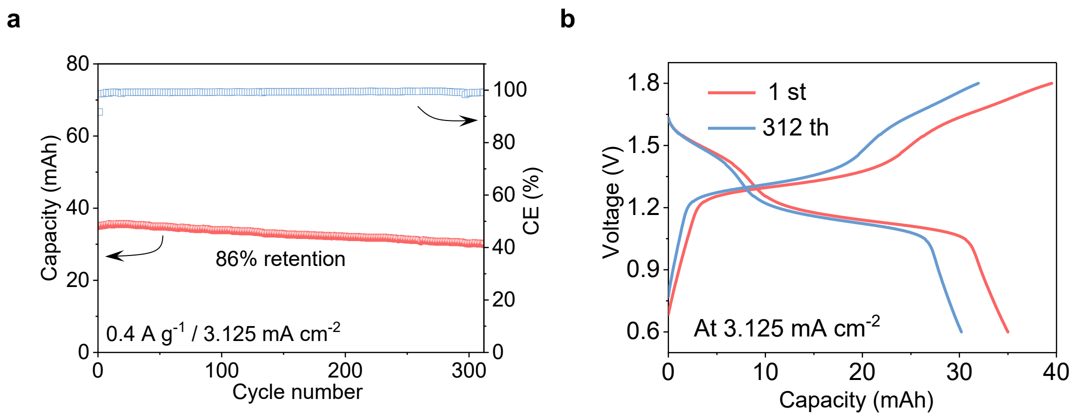

Fig. S60 Electrochemical tests of pouch cell. **a**, Long-term cycling performance and **b**, GCD curves of pouch cell with a theoretical capacity of 35 mAh at 0.4 A g^{-1}.

Table S1 Hammett parameters for different functional groups.

Electron-withdrawing group		
Structural formula	σ（substitution）	σ（intermediate）
$-NO_2$	+0.82	+0.71
-CN	+0.66	+0.56
$-SO_3H$	+0.69	+0.39
$-CF_3$	+0.43	+0.43
-COOH	+0.45	+0.37
-COOR	+0.45	+0.37
$-CONH_2$	+0.28	+0.15
$-COCH_3$	+0.51	+0.37
-F	+0.06	+0.34
-Cl	+0.23	+0.37
-Br	+0.23	+0.39
-I	+0.28	+0.35

Electron-donating group		
Structural formula	σ（substitution）	σ（intermediate）
$-NH_2$	-0.66	-0.16
$-OCH_3$	-0.27	+0.12
-OH	-0.37	+0.12
$-CH_3$	-0.17	-0.07
$-C_2H_5$	-0.15	-0.07
-Ph	-0.01	-0.01
$-C_4H_4O$	-0.01	-0.01

Table S2 Specific power / energy comparison of Zn||I_2 batteries with other systems.

Systems	References	Specific power (W kg^{-1})	Specific energy (Wh kg^{-1})
I^-/I^0	Ref. 36	553/1118/2763/5755/15064	208/201/188/160/142
Br^-/Br^0	Ref. 37	232/444/728/1129/1700	376/287/210/176/100
S^{2-}/S^0	Ref. 38	91/223/346/483	385/185/99/40
S^0/S^{4+}	Ref. 39	67/381/890/1905/2980	359/281/248/212/183
Se^-/Se^+	Ref. 40	771/1575/3127/7806/15500	95/82/76/70/63
Se^{2-}/Se^{4+}	Ref. 41	100/221/353/915/1277	376/315/243/137/107
Te^{2-}/Te^0	Ref. 42	81/243/470/808/1590	232/225/186/145/86
T^0/Te^{4+}	Ref. 43	336/840/1352/2465	389/348/232/141
$ZnBr_2$		967/1761/2569/4189/8189/15631	336/301/285/265/237/205
BrAce		1244/2476/3738/6169/12515/17202	439/423/415/390/362/320

Table S3 Enthalpies and Gibbs free energies of all reaction substrates for four-electron iodine conversion and hydrolysis reactions.

Reaction substrate	H (Hartree)	G (Hartree)
Br^0	-2574.14799	-2574.16718
I^0	-297.609488	-297.629351
I^-	-297.719869	-297.739078
I_2	-595.273368	-595.302861
Br^-	-2574.268609	-2574.28715
I_3^-	-893.044237	-893.082507
Ace	-209.13012	-209.163662
Ace^-	-208.478745	-208.511977
C-Br-I	-7523.162881	-7523.21713
Br···C···I	-7523.106936	-7523.15697
C-I-Br	-7523.150499	-7523.20344
I-Br	-2871.823919	-2871.82392
H_2O	-76.400765	-76.422834
HIO	-373.402966	-373.431852
HBr	-2574.781148	-2574.80368

Table S4 Comparative data of iodine utilization between the BrAce system and other systems under high loading and high current density conditions.

Systems	References	Loading (mg cm^{-2})	Current density (mA cm^{-2})	Iodine utilization (%)
$I^-/I^0/I^+$	Ref. 7	0.80	6.40	69.9
$I^-/I^0/I^+$	Ref. 12	5.00	5.00	76.1
$I^-/I^0/I^+$	Ref. 13	10.00	5.00	73.4
I^-/I^0	Ref. 56	7.82	1.56	44.1
I^-/I^0	Ref. 57	20.50	3.40	50.1
I^-/I^0	Ref. 58	14.10	2.80	86.5
I^-/I^0	Ref. 59	15.33	1.53	89.1
$I^-/I^0/I^+$	Ref. 60	3.00	1.50	54.5
$I^-/I^0/I^+$	Ref. 61	2.00	2.00	83.1
This work		8.60	3.44	80.1
This work		14.50	5.80	75.2
This work		24.00	9.60	74.0

Table S5 Cost comparison between the BrAce system and other systems.

Systems	References	Cost (US$ mL^{-1})
$ZnCI_2$-LiCI-AN	Ref. 3	0.837
30 M $ZnCl_2$	Ref. 4	0.455
10 M $ZnBr_2$	Ref. 5	0.132
$ZnCl_2$-TEACl	Ref. 6	0.339
$Zn(CIO_4)_2$-NA-MSM	Ref. 7	0.177
$Zn(OTF)_2$-Urea	Ref. 8	0.366
FeSA-NC	Ref. 13	0.152
$Zn(OAC)_2$-LiCl	Ref. 21	0.361
$ZnCI_2$-LiOTF	Ref. 62	1.033
This work		0.095

Supplementary References

1. Feng, Z. et al. Reducing dead species by electrochemically-densified cathode-interface-reaction layer towards high-rate-endurable Zn||I-Br batteries. *Angew. Chem. Int. Ed.* **64**, e202416755 (2025).
2. Liu, T. et al. Practical four-electron zinc-iodine aqueous batteries enabled by orbital hybridization induced adsorption-catalysis. *Sci. Bull.* **69**, 1674-1685 (2024).
3. Zong, W. et al. Dynamical janus interface design for reversible and fast-charging zinc–Iodine battery under extreme operating conditions. *J. Am. Chem. Soc.* **146**, 21377-21388 (2024).
4. Liang, G. et al. Development of rechargeable high-energy hybrid zinc-iodine aqueous batteries exploiting reversible chlorine-based redox reaction. *Nat. Commun.* **14**, 1856 (2023).
5. Li, W. et al. Designing ternary hydrated eutectic electrolyte capable of four-electron conversion for advanced Zn–I_2 full batteries. *Energy Environ. Sci.* **16**, 4502-4510 (2023).
6. Guan, D. et al. π-d conjugated coordination mediated catalysis for four-electron-transfer fast-charging aqueous zinc-iodine batteries. *Matter* **8**, 101932 (2025).
7. Wang, C. et al. Inside cover: activating and stabilizing a reversible four electron redox reaction of I^-/I^+ for aqueous Zn-iodine battery. *Angew. Chem. Int. Ed.* **63**, e202409271 (2024).
8. Hu, T. et al. Development of inverse-opal-structured charge-deficient Co_9S_8@nitrogen-doped-carbon to catalytically enable high energy and high power for the two-electron transfer I^+/I^- Electrode. *Adv. Mater.* **36**, 2312246 (2024).
9. Kang, J. et al. Electron-outflowing heterostructure hosts for high-voltage aqueous zinc-iodine batteries. *Energy Storage Mater.* **68**, 103367 (2024).
10. Liu, T. et al. Triflate anion chemistry for enhanced four-electron zinc–iodine aqueous batteries. *Chem. Commun.* **60**, 7447-7450 (2024).
11. Han, W., Zhao, J. & Li, X. Ternary chloride-free electrolyte design for highly efficient aqueous zinc–iodine batteries with four-electron conversion. *Inorg. Chem. Front.* **11**, 5376-5383 (2024).
12. Wang, M. et al. Bidentate coordination structure facilitates high-voltage and high-utilization aqueous Zn−I_2 batteries. *Angew. Chem. Int. Ed.* **63**, e202404784 (2024).
13. Zou, Y. et al. A four-electron Zn-I_2 aqueous battery enabled by reversible $I^-/I_2/I^+$ conversion. *Nat. Commun.* **12**, 170 (2021).
14. Jiang, P. et al. Stabilized four-electron aqueous zinc–iodine batteries by quaternary ammonium complexation. *Chem. Sci.* **15**, 3357-3364 (2024).
15. Li, X. et al. Perovskite cathodes for aqueous and organic iodine batteries operating under one and two electrons redox modes. *Adv. Mater.* **36**, 2304557 (2024).

16. Chen, S. et al. Solid interhalogen compounds with effective Br^0 fixing for stable high-energy zinc batteries. *Angew. Chem. Int. Ed.* **62**, e202301467 (2023).

17. Liu, T. et al. Aqueous electrolyte with weak hydrogen bonds for four-electron zinc–iodine battery operates in a wide temperature range. *Adv. Mater.* **36**, 2405473 (2024).
