# Supplementary material for: Carbon-halogen bond substitution enables high-utilization four-electron iodine redox in noncorrosive dilute electrolytes
Source: Nat Commun. 2026 Feb 21;17:3048. doi: 10.1038/s41467-026-69743-z (PMC13039508; doi:10.1038/s41467-026-69743-z)
Supplement: Supplementary file 2 — Description of Additional Supplementary Files [file 41467_2026_69743_MOESM2_ESM.pdf]

Description of Additional Supplementary Files

Supplementary Data 1: Optimized atomic coordinate for R-CH_2-Br (BrAce) (R, NH_2-(C=O)-)
Supplementary Data 2: Optimized atomic coordinate for R-CH_2-Br···I^0
Supplementary Data 3: Optimized atomic coordinate for R-(I^0···(CH_2) ···Br)
Supplementary Data 4: Optimized atomic coordinate for R-CH_2-$I^{(+)}$-Br
Supplementary Data 5: Optimized atomic coordinate for IBr···H_2O
Supplementary Data 6: Optimized atomic coordinate for IBr···H_2O (TS: transition state)
Supplementary Data 7: Initial configuration of HIO
Supplementary Data 8: Initial configuration of HBr
Supplementary Data 9: Initial configuration of R-CH_2-$I^{(+)}$-Br···H_2O
Supplementary Data 10: Initial configuration of R-CH_2-$I^{(+)}$-Br···H_2O (TS)
Supplementary Data 11: Initial configuration of R-CH_2-I (IAce)
